# Integrating evidence-based PTSD treatment into intensive eating disorders treatment: a preliminary investigation

**DOI:** 10.1007/s40519-022-01500-9

**Published:** 2022-11-19

**Authors:** Kimberly Claudat, Erin E. Reilly, Alexandra D. Convertino, Julie Trim, Anne Cusack, Walter H. Kaye

**Affiliations:** 1 Department of Psychiatry, University of California, San Diego, 4510 Executive Drive, Suite 315, San Diego, CA 92121 USA; 2 Department of Psychiatry, University of California, San Francisco, USA; 3 San Diego State University, University of California San Diego Joint Doctoral Program in Clinical Psychology, San Diego, USA

**Keywords:** Eating disorders, Treatment, Post-traumatic stress disorder, Partial-hospital program, Co-occurring disorders

## Abstract

**Purpose:**

Given data suggesting common co-occurrence and worse outcomes for individuals with eating disorders (EDs) and post-traumatic stress disorder (PTSD), it is critical to identify integrated treatment approaches for this group of patients. Past work has explored the feasibility and initial efficacy of intervention approaches that draw on evidence-based treatments for both EDs and PTSD; however, this work remains limited in scope. In the current study, we explored the feasibility and naturalistic outcomes of PTSD treatment delivered within the context of intensive ED treatment.

**Method:**

Participants were 57 adult men and women with DSM-5 EDs and comorbid PTSD who completed a course of either Prolonged Exposure (PE; *n* = 22) or Cognitive Processing Therapy (CPT; *n* = 35) (M_sessions_ = 10.40; SD = 5.13) and weekly validated measurements of clinical symptoms while enrolled in ED programming.

**Results:**

Multi-level models for PTSD symptoms indicated a significant linear effect of time, such that participants demonstrated significant decreases over time in PTSD symptoms, regardless of treatment modality.

**Conclusion:**

Our preliminary investigation provides support for the feasibility and efficacy of an integrated approach to treating EDs and PTSD. It is critical for future work to undertake randomized tests of this integrated approach using large, heterogeneous samples.

**Level of evidence:**

Level IV, multiple time series with intervention

**Supplementary Information:**

The online version contains supplementary material available at 10.1007/s40519-022-01500-9.

## Introduction

Eating disorders (EDs), including anorexia nervosa, bulimia nervosa, and binge eating disorder, are associated with significant increases in risk for premature mortality [12], severe medical complications [40], and high levels of distress and functional impairment [4, 5, 8]. Data from randomized controlled trials of evidence-based treatments for EDs suggest that many patients do not achieve full remission [7, 24], with a significant portion of patients demonstrating a chronic course of illness [22, 33]. One common predictor of poor outcome in EDs includes the presence of co-occurring psychopathology, particularly post-traumatic stress disorder (PTSD) [30, 31]. There is thus an imperative to better understand this overlap and identify adjunctive treatments for these populations.

Research suggests that EDs and PTSD are highly comorbid and may represent a high-risk group for poor outcomes [30, 31]. Data suggest that nearly all individuals with EDs in community samples have experienced trauma [27, 35], and between 25 and 40% of individuals with a diagnosed ED meet criteria for PTSD or endorse clinically significant symptoms of PTSD [11, 27]. Rates of PTSD are even higher among individuals in ED treatment, particularly in higher levels of care, with one study showing 52% of women hospitalized for an ED reported PTSD or symptoms consistent with PTSD [16].

Importantly, individuals with this comorbidity (ED-PTSD) have higher severity of illness, greater rates of psychiatric comorbidity, and worse response to ED treatment [6, 30, 31, 35]. Because existing evidence-based approaches to treating EDs [9, 25] or PTSD [19, 20, 29] generally do not include guidance on how to address these overlapping symptom presentations, therapists treating patients with EDs and PTSD anecdotally report a “whack-a-mole” phenomenon throughout intervention, such that direct intervention on one set of symptoms often results in an exacerbation of the other [35]. Altogether, there is a dire need to improve outcomes for patients who experience both PTSD and EDs through the development and testing of integrated intervention approaches.

Despite data highlighting the important link between EDs and PTSD, and a call for integrated treatments of these disorders, there are limited guidelines on how to integrate the treatment of PTSD into ED treatment. Consequently, clinicians and researchers have aimed to apply the principles of established evidence-based treatments for PTSD into ED treatment. Cognitive Processing Therapy (CPT) [29], and Prolonged Exposure (PE) [14] are cognitive-behavioral therapies that have the strongest evidence base of all the PTSD treatment protocols for adults and are considered first-line treatments [39]. Therefore, these evidence-based treatments represent promising protocols to integrate into ED treatment.

First, CPT is approximately 12 sessions, 45–60 min in length, typically administered weekly or twice a week. Based on cognitive-behavioral conceptualizations of PTSD [29], the CPT therapist facilitates the cognitive and emotional processing of the traumatic event and its impact on the individual. The focus is on identifying and modifying “stuck points,” or maladaptive beliefs related to why the traumatic event occurred, as well as beliefs related to safety, power/control, esteem, intimacy, and trust. To date, only a handful of studies exists that examine integrated CPT for ED-PTSD, all by the same research group [37]. This integrated treatment has been manualized by combining CPT with ED interventions based on CBT-E [9]. The initial study on this treatment found preliminary support for the efficacy of integrated CBT for ED-PTSD in a sample of 10 patients, with the majority of participants experiencing improvement in depression, anxiety, ED symptoms, and PTSD symptoms. More recently, a larger randomized controlled trial with 48 patients reaffirmed the potential of this approach, suggesting that integrated CPT and ED treatment resulted in greater decreases in PTSD symptoms than ED treatment alone [38]. Therefore, while the majority of work thus far has been completed by one research group, preliminary findings are extremely promising regarding the effectiveness and feasibility of integrating CPT with ED treatment.

Second, PE is typically completed in 9–15 weekly individual therapy sessions, approximately 90 min in length. Based on Emotional Processing Theory [15], the client participates in in-vivo and imaginal exposures to approach trauma-related memories, feelings, and triggers that have been avoided. There are currently no data on the integration of PE into ED treatment. However, research on DBT-PE (PE integrated into DBT for multidiagnostic and severe cases) indicates that it is effective in significantly reducing PTSD symptoms, depression, and anxiety [17, 18]. More research on the integration of PE into ED treatments is necessary to understand its efficacy with this patient population.

## Current study

Literature on the integration of evidence-based PTSD treatments into existing ED treatment protocols is scarce. Therefore, to build upon previous research, the present study examined the feasibility and naturalistic treatment outcomes for patients with PTSD presenting to intensive ED treatment, who completed a course of CPT or PE during their ED treatment stay. Given previous research findings, we tested the hypothesis that participants would endorse significant decreases in PTSD symptoms following PTSD treatment. Furthermore, we hypothesized that PTSD treatment completion would also be associated with a significant decrease in eating pathology, depression, and anxiety.

## Method

### Participants and procedures

Data for the current study was based on a retrospective chart review of 57 adult patients who were admitted to the [blinded for peer review] partial hospitalization/day treatment program for EDs between November 2016 and July 2021 and participated in trauma-based treatment for PTSD. For patients who had multiple admissions (*n* = 6), data from their first admission with trauma treatment was used. Participants completed clinical interviews and self-report questionnaires at admission and discharge. ED and PTSD diagnoses were assessed using either the MINI Neuropsychiatric Interview (MINI) [32] or the Structured Clinical Interview for DSM-5 (SCID-5) [13] administered by trained, bachelor’s-level research assistants who were supervised by licensed clinical psychologists. For more details, please see the Supplement. This study received ethical approval through the [blinded for peer review] Institutional Review Board.

#### ED treatment

The partial hospital and intensive outpatient programs are primarily based in dialectical behavior therapy (DBT), including skills coaching, weekly DBT skills groups, weekly individual therapy, and weekly DBT consultation team meetings. In addition to DBT skills groups, patients also attend other therapeutic groups including enhanced cognitive behavior therapy for EDs [9], acceptance and commitment therapy [26], process groups, nutrition, and goal setting. Further description of the program can be found in [blinded for peer review].

#### Trauma-based treatment

As detailed previously, the two PTSD treatments administered were PE (*n* = 22) and CPT (*n* = 35). In accordance with Va/DOD (2013) PTSD clinical practice guidelines, selection of evidence-based trauma treatment protocol was based on patient preference and clinician training and expertise [38]. Patient eligibility to begin trauma treatment was determined by adapting previously recommended guidelines both for PE with multidiagnostic, complex cases, and CPT for ED [17, 38]. Generally, PTSD had to be identified as a top treatment target by clinical team and patient, patients were expected to be willing to engage in PTSD treatment, nutritionally and weight stable (minimum 85% IBW), as well as behaviorally stable, with no imminent safety concerns or life-threatening behaviors (See Supplement for more detailed information). Furthermore, since treatment occurred in a clinical setting, treatment length varied by patient needs. In addition to self-report questionnaires of ED symptoms and other psychopathological symptoms measured at intake and discharge, assessment of PTSD symptoms was administered each session or every other session depending on treatment protocol recommendations.

### Measures

#### PTSD symptoms

PTSD symptoms were measured by the PTSD Checklist for DSM-5 (PCL-5) [3]. The PCL-5 is a 20-item self-report measure that assesses the degree to which an individual has been bothered by DSM-5 PTSD symptoms in the past week in relation to their most distressing traumatic event (i.e., the “index trauma” they chose to work on). Items are rated on a five-point scale from 0 (*not at all*) to 4 (*extremely*) and total scores range from 0 to 80, with higher scores indicating greater severity of PTSD symptoms. As noted previously, the PCL-5 was administered every session for participants completing CPT, and every other session for those receiving PE.

#### Secondary outcome measurements

ED symptoms, ED-related psychosocial impairment, depression, and anxiety were all measured at intake to the clinic and discharge.

#### ED symptoms

ED symptoms were measured by the Eating Disorder Examination Questionnaire (EDE-Q) [10]. The EDE-Q is a 31-item self-report questionnaire that assesses the presence and frequency of eating disorder symptoms over the last 28 days. Items are rated on a seven-point scale from 0 (*no days*) to 6 (*every day*). Higher scores indicate greater frequency of eating disorder symptoms. The EDE-Q produces four subscales (Dietary Restraint, Shape Concern, Weight Concern, and Eating Concern), as well as a global score. For the purposes of this study, we used the global score as an indicator of overall ED symptoms.

#### ED-related psychosocial impairment

ED-related psychosocial impairment was measured by the Clinical Impairment Assessment Questionnaire (CIA) [5]. The CIA is a 16-item self-report measure that assesses the severity of impairment over the last 28 days. Items are rated on a four-point scale from 0 (*not at all*) to 4 (*a lot*) and total scores range from 0 to 48, with higher scores indicating greater severity of psychosocial impairment.

#### Anxiety symptoms

Anxiety symptoms were measured by the trait subscale of the State-Trait Anxiety Inventory for Adults (STAI) [34]. The trait subscale of the STAI has 20 items for assessing general tendency toward anxious symptoms. Items are rated on a four-point scale from 1 (*almost never*) to 4 (*almost always*) and the scores range from 20 to 80, with higher scores indicating greater tendency towards anxiety.

#### Depression symptoms

Depression symptoms were measured by the Beck Depression Inventory (BDI) [2] and the Patient Health Questionnaire-9 (PHQ-9) [23]. The BDI is a 21-item self-report questionnaire with items rated on a four-point scale from 0 to 3, with higher scores indicating greater depressive symptoms. The PHQ-9 is a 9-item self-report questionnaire with items rated on a four-point scale from 0 (*not at all*) to 3 (*nearly every day*), with higher scores indicating greater depressive symptoms. Due to changes in assessment protocols, some participants were administered the BDI (*n* = 22), and some were administered the PHQ-9 (*n* = 36). To compare across measures, *z*-scores were calculated for the BDI and PHQ-9 separately. Changes in depressive symptoms are therefore reported in terms of *z*-scores.

### Analysis plan

#### Primary analyses

Analyses were conducted using the intent-to-treat sample with all participants that had completed at least one session of treatment. Descriptive analyses were run using Statistical Package for the Social Sciences (SPSS; version 28). To explore changes in PTSD symptoms, linear mixed effects analyses were conducted using R software [28] using the “lme4” package [1] with full information maximum likelihood estimation to account for missing data. The model examining the effect of time on PTSD symptoms included the fixed effects of time, as well as random effects of participant and time. Meaningful change was defined as change in PCL-5 score exceeding the reliable change index (RCI) [21] calculated from the initial validation sample (*RCI* = 10; [3, 40]).

#### Supplemental, exploratory analyses

Given attrition, only a subset of participants (*n* = 38) completed both admission and discharge self-report questionnaires (i.e., secondary outcomes). All of the participants completed PTSD symptom reports. Differences in self-report symptoms between admission and discharge for secondary outcomes were calculated using paired-samples *t*-tests in SPSS. Clinically meaningful change for these outcomes was defined as exceeding the RCI as follows: EDE-Q Dietary Restraint RCI = 1.95; EDE-Q Shape Concern RCI = 1.35; EDE-Q Weight Concern RCI = 1.85; EDE-Q Eating Concern RCI = 2.15; EDE-Q Global Score = 0.89; CIA RCI = 8.10; STAI Trait = 11.10 [8]. RCI of Depression symptoms were not examined due to the two different measures administered. Additionally, an exploratory analysis with this subset of participants was conducted using linear mixed effects analyses in R with the “lme4” package, with full information maximum likelihood estimation. This analysis explored moderators of the PTSD time trajectory, including illness duration, ED-related psychosocial impairment, depression symptoms, global eating pathology, and anxiety symptoms at admission.

## Results

### Primary analyses

Of 77 individuals who were diagnosed with PTSD at admission to the treatment program, 57 participants received PTSD treatment. See Table 1 for full demographics. In total, 49 out of 57 participants were considered PTSD treatment completers, such that the patient and therapist mutually decided that the patient had achieved meaningful change and would discontinue treatment. The maximum number of sessions administered was 24, but participants most typically completed 12 sessions (*M* = 10.40, SD = 5.13). Results from the multi-level model revealed that the average PCL-5 score at the first session was 50.83 (SE = 1.94). Analyses showed a main effect of time, such that overall, PTSD symptoms decreased significantly over the course of treatment (*b* = − 2.40; SE = 0.24). See Table 2 for full results.

**Table 1:** Sociodemographic characteristics of sample by trauma treatment type

Sociodemographic characteristic	Cognitive processing	Prolonged exposure
Sample *n*	35	22
Age of onset (*M*, SD)	13.55 (4.86)	13.68 (5.60)
Age at admit (*M*, SD)	25.75 (8.32)	30.48 (11.45)
Body mass index at admit (*M*, SD)	21.92 (5.72)	24.75 (9.69)
Body mass index at discharge (*M*, SD)	23.68 (5.17)	26.66 (9.07)
Years of education (*M*, SD)	14.04 (1.67)	15.26 (2.20)
Length of eating disorder treatment in days (*M*, SD)	122.63 (50.65)	137.32 (41.89)
Number of binge episodes at admit (*M*, SD)	7.30 (15.02)	9.14 (17.47)
Number of binge episodes at discharge (*M*, SD)	0.67 (1.41)	1.44 (2.71)
Number of binge days at admit (*M*, SD)	5.41 (9.21)	4.52 (8.16)
Number of binge days at discharge (*M*, SD)	0.67 (1.86)	0.95 (1.84)
PCL-5 score at beginning of treatment (*M*, SD)	51.17 (15.25)	45.86 (15.42)
PCL-5 score at end of treatment (*M*, SD)	31.31 (22.23)	28.27 (15.89)
Number of PTSD sessions (*M*, SD)	9.69 (5.06)	11.55 (5.16)
Diagnosis at admit (*n*, %)		
Anorexia nervosa, restricting type	8 (22.9)	5 (22.7)
Anorexia nervosa, binge/purge type	6 (17.1)	4 (18.2)
Bulimia nervosa	10 (28.6)	6 (27.3)
Binge eating disorder	1 (2.9)	0 (0.0)
Avoidant-restrictive food intake disorder	2 (5.7)	2 (9.1)
Other specified feeding and eating disorder	8 (22.9)	5 (22.7)
Sex (*n*, %)		
Male	3 (8.6)	2 (9.1)
Female	32 (91.4)	20 (90.9)
Gender (*n*, %)^a^		
Man	3 (8.6)	2 (9.1)
Woman	27 (77.1)	19 (86.4)
Genderqueer/gender non-conforming	1 (2.9)	1 (4.5)
Race (*n*, %)		
White	31 (88.6)	19 (86.4)
Native American/Alaska Native	1 (2.9)	0 (0.0)
Asian	0 (0.0)	2 (9.1)
Other	3 (8.6)	1 (4.5)
Ethnicity (*n*, %)		
Hispanic/Latinx	7 (20.0)	8 (36.4)
Not Hispanic/Latinx	28 (80.0)	16 (63.6)
Medication^b^		
Selective serotonin reuptake inhibitors/antidepressants at admit	24 (68.6)	18 (85.7)
Selective serotonin reuptake inhibitors/antidepressants at discharge	22 (62.9)	16 (76.2)
Atypical antipsychotics at admit	10 (28.6)	4 (19.0)
Atypical antipsychotics at discharge	10 (28.6)	2 (9.5)
Mood stabilizer at admit	7 (20.0)	6 (28.6)
Mood stabilizer at discharge	8 (22.9)	7 (33.3)
Alcohol/substance use medication at admit	1 (2.9)	2 (9.5)
Alcohol/substance use medication at discharge	0 (0.0)	1 (4.8)
Anxiety medication at admit	6 (17.1)	9 (42.9)
Anxiety medication at discharge	7 (20.0)	6 (28.6)
Attention-deficit/hyperactivity disorder medication at admit	4 (11.4)	1 (4.8)
Attention-deficit/hyperactivity disorder medication at discharge	3 (8.6)	1 (4.8)
Sleep medication at admit	4 (11.4)	2 (9.5)
Sleep medication at discharge	3 (8.6)	2 (9.5)
Other medication at admit	3 (8.6)	1 (4.8)
Other medication at discharge	3 (8.6)	1 (4.8)

*SD* standard error, *M* mean

^a^Four participants (11.4%) assigned to cognitive processing therapy did not report gender

^b^Of the participants assigned to cognitive processing therapy, six participants were missing medication information at admit and seven were missing medication information at discharge. Of the participants assigned to prolonged exposure, one participant was missing medication information at admit and two were missing medication information at discharge

**Table 2:** Results of linear mixed model with time predicting post-traumatic stress disorder symptoms

Fixed effects	Estimate	95% Confidence interval	*p*
Intercept	50.83	47.34; 54.96	< .001
Time	−2.40	−2.91; −1.96	< .001

Out of 57 participants who began trauma treatment, 40 (70.2%) achieved clinically meaningful change in their PTSD symptoms at the end of trauma treatment, as compared to 17 (29.8%) that did not.

### Supplemental analyses

In terms of change for non-PTSD psychological symptoms, there were significant changes in all measured psychological symptoms from admission to discharge. Change in dietary restraint (*M* = 2.47; SD = 1.66), shape concern (*M* = 1.76; SD = 1.44), weight concern (*M* = 1.91; SD = 1.55), global eating pathology (*M* = 2.04; SD = 1.30), ED-related psychosocial impairment (*M* = 18.27; SD = 9.59), and anxiety symptoms (*M* = 13.33; SD = 13.93) met or exceeded the RCI, meaning that clinically significant change was achieved (see “methods” section for RCI threshold information). Eating concern (*M* = 2.01; SD = 1.51) did not exceed this threshold. See Table 3 for full results.

**Table 3:** Change in psychological symptoms at admission and discharge

Psychological symptom (*M*, SD)	Admission	Discharge	*t*	Cohen’s *d*
Dietary restraint	3.44 (1.86)	0.97 (1.14)	9.20	1.49
Eating concerns	3.32 (1.56)	1.30 (1.33)	8.25	1.34
Shape concerns	4.76 (1.65)	3.00 (1.78)	7.53	1.22
Weight concerns	4.47 (1.59)	2.56 (1.90)	7.60	1.23
Global eating pathology	4.00 (1.46)	1.96 (1.41)	9.71	1.58
Eating disorder-related psychosocial impairment	34.50 (8.70)	16.24 (11.25)	10.61	1.91
Anxiety symptoms	66.89 (7.82)	53.55 (13.52)	5.90	0.96
Depression symptoms	0.47 (0.79)	− 0.51 (0.93)	6.50	1.05

All differences were statistically significant (*p* < .001). For these analyses, *n* = 38 due to missing data. For context of Cohen’s *d*, 0.2 is considered a ‘small’ effect size, 0.5 is considered a ‘medium’ effect size, and 0.8 is considered a ‘large’ effect size

Results from the multi-level model revealed that change in PTSD symptoms was moderated by admission ED-related psychosocial impairment (*b* = − 0.15; SE = 0.06) and eating pathology symptoms (*b* = 0.75; SE = 0.23). Individuals with greater eating disorder symptoms at baseline (1SD above the mean) have a steeper trajectory of change over the course of treatment (*b* = − 3.41; SE = 0.54), such that these individuals benefit with greater reductions in PTSD symptoms as compared to those with lower eating disorder symptoms at baseline (1SD below the mean; *b* =− 1.32; SE = 0.36). Individuals with lower ED-related psychosocial impairment (1SD below the mean) have a steeper trajectory of change over the course of treatment (*b* = − 3.68; SE = 0.72), such that these individuals benefit with greater reductions in PTSD symptoms as compared to those with higher ED-related psychosocial impairment as admission (1SD above the mean; *b* = − 1.06; SE = 0.43). See Table 4 for full results.

**Table 4:** Results of linear mixed model with moderators of time predicting post-traumatic stress disorder symptoms

Fixed effects	Estimate	95% Confidence interval	*p*
Intercept	46.21	41.94; 50.34	< .001***
Time	− 1.99	− 2.44; − 1.54	< .001***
Illness duration	0.67	0.33; 1.01	.001**
ED-related psychosocial impairment	1.23	0.59; 1.89	.001**
Depression symptoms	− 1.30	− 6.42; 3.80	.642
Eating pathology symptoms	− 0.94	− 4.61; 2.90	.648
Anxiety symptoms	− 0.32	− 0.95; 0.31	.352
Time × illness duration	− 0.01	− 0.05; 0.02	.436
Time × ED-related psychosocial impairment	− 0.11	− 0.19; − 0.03	.026*
Time × depression symptoms	0.58	0.03; 1.12	.073
Time × eating pathology symptoms	0.44	0.01; 0.86	.073
Time × anxiety symptoms	− 0.07	− 0.14; 0.00	.097

*p* < .001***, *p* < .01**,  *p* < .05*

## Discussion

The present study examined treatment outcomes associated with evidence-based PTSD treatment delivered within the context of a DBT partial hospital/intensive outpatient program for EDs. Results indicated that a group of 57 individuals who engaged in either CPT or PE for PTSD reported significant reductions in PTSD symptoms over the course of treatment, with approximately 70% experiencing reliable change in symptoms over time. Additionally, participants demonstrated a reduction in global eating pathology, ED-related psychosocial impairment, anxiety symptoms, and depressive symptoms over the general course of ED treatment. While our study possesses several limitations, including the lack of a control group and notable attrition for secondary outcomes, our data nonetheless provide further support for calls to explore integrated ED and PTSD treatment.

### What is already known on this subject?

Research indicates that EDs and PTSD are highly comorbid, and that individuals with this comorbidity may represent a high-risk group for poor treatment outcomes. However, data on the treatment of ED-PTSD are limited, and there is no consensus for how and when to integrate PTSD treatment into ED treatment.

### What this study adds?

Results from the main study analyses are consistent with study hypotheses and preliminary findings from other clinical groups, providing preliminary evidence consistent with other work on integrating evidence-based PTSD treatments into ED treatment [6, 36, 37]. Particularly given the lack of formal guidance for approaching treatment planning for individuals with EDs and comorbidities, our findings provide tentative support for positive outcomes associated with targeting symptoms of both co-occurring disorders while in ED treatment. Without integrated care, therapists treating both ED and PTSD often observe the aforementioned “whack-a-mole” phenomenon, which likely results from ignoring maintaining factors for both the ED and PTSD, and our findings suggests that future work must continue to test whether this approach improves treatment outcomes related to ED symptomatology, PTSD symptoms, and overall psychosocial functioning.

Secondary, exploratory analyses in a subset of the sample suggested that individuals with lower levels of psychosocial impairment demonstrated a greater decrease in symptoms over time. While these results must be interpreted cautiously given the small sample size and attrition in study measures, it may be the case that the finding regarding lower levels of impairment is related to capacity for treatment engagement. Individuals experiencing greater impairment associated with their ED at baseline may not have been able to engage as fully in treatment procedures for PTSD due to other stressors and/or obligations, resulting in less clinical change. However, this interpretation remains speculative and must be tested by future research. Our moderation analyses also revealed an effect of baseline ED symptoms, such that those with greater ED symptoms at baseline demonstrated greater change over time. This effect may simply reflect regression to the mean, such that individuals with elevated symptoms tend to show greater changes over time. Given robust positive associations between ED symptoms and PTSD symptoms, it is also possible that individuals with greater ED symptoms may also have higher PTSD symptoms and are more motivated for change. Again, this possibility should be evaluated directly in the future.

### Strengths and limits

The current findings help expand the literature and our understanding of the ED-PTSD population. This study’s naturalistic study design provides evidence for the feasibility of integrating evidence-based PTSD protocols, without adaptation, into an intensive ED treatment setting for patients presenting with co-occurring disorders and high levels of clinical impairment. Of note, this study is not without its limitations. First, participants were drawn from a treatment-seeking sample presenting at a higher level of care. As such, results may not be representative of individuals with ED-PTSD in the broader community. Second, this is a relatively small sample size, and consisted of mostly women, thus reducing potential to generalize the findings to other populations. Third, it is important to note that the multi-component structure of the treatment program, while generally representative of similar intensive programs for EDs, limits our ability to attribute observed changes in clinical symptoms to PTSD treatment, versus other components of our treatment approach. Relatedly, and most importantly, this was not a controlled study; therefore, future randomized controlled trials are needed to examine outcomes to determine the efficacy of these specific treatments to ED-PTSD, as well as other evidence-based trauma treatments.

In summary, the present model and preliminary data highlight the feasibility and potential efficacy of integrating evidence-based treatments for PTSD, such as PE and CPT, into ED treatment. More research is necessary to elucidate best practices and improve care for individuals with co-occurring ED-PTSD.

## Supplementary Information

Below is the link to the electronic supplementary material.

Supplementary file 1: (DOCX 23 KB)
