# Supplementary material for: Integrating evidence-based PTSD treatment into intensive eating disorders treatment: a preliminary investigation
Source: Eat Weight Disord. 2022 Nov 19;27(8):3599–607. doi: 10.1007/s40519-022-01500-9 (PMC9803734; doi:10.1007/s40519-022-01500-9)
Supplement: Supplementary file 1 — (DOCX 23 KB) [file 40519_2022_1500_MOESM1_ESM.docx]

**Supplement**

**Interviewing Procedures**

Adult participants completed either the Structured Clinical Interview for the DSM-5 (SCID-5; (First et al., 2015) or the MINI Neuropsychiatric Interview 7.0 upon admission (Sheehan et al., 1998). Assessors reviewed each case within weekly meetings with other assessors and a senior postdoctoral fellow and/or licensed clinical psychologist, both with expertise in diagnostic assessment. Assessors reviewed each item on the semi-structured interview and made a case for which criteria were appropriately endorsed based on patient responses. The assessor then made the case for which diagnosis DSM-5 diagnosis would best fit the symptoms endorsed or presented questions to the team if they were deciding between two diagnoses. After group and supervisor feedback, a final diagnosis was confirmed. Records on diagnostic decisions were kept over the course of consensus meetings to ensure inter-patient consistency.  If insufficient details were gathered on any of the assessment items (e.g., not enough information to determine whether an episode of eating would be considered objectively large), the assessor was directed to follow-up directly with the patient to get additional details and bring this information back to the next meeting for review. All loss-of-control eating episodes were reviewed in consensus and compared to standards established by the interview for being objectively larger than what most people would consume under similar circumstances. When ambiguous, the consensus team voted whether the episode was objectively large or not, with final input from supervisors.

**Readiness Criteria for Trauma Treatment**

The following criteria were utilized to establish readiness for the start of trauma treatment.

Safety

- No suicide attempts for at least 2 months
- No self-harm for at least 1 month
- Not at imminent risk of suicide
- Ability to control life-threatening behavior in the presence of cues for those behaviors (e.g., trauma reminders; thoughts and emotions related to trauma)

Other criteria

- No serious therapy-interfering behavior
- PTSD is the highest priority target and the client wants PTSD treatment right now
- Demonstrates ability and willingness to experience intense emotions without escaping

Maladaptive behaviors

- *Eating disorder*
  - Greater than 85% Ideal Body Weight (IBW)
  - Behaviors occur no more than a few times a week at most, and showing consistent reduction in behavior frequency
  - Patient can commit to not using an ED behavior in response to trauma-related emotion for at least 30 minutes
- *Substance use*
  - If patient is using a harm-reduction model, patient has been successful in reducing frequency or intensity of use
  - Patient has demonstrated that s/he can tolerate intense emotions without using substances
